# Inverse Relationship between PSA and IL-8 in Prostate Cancer: An Insight into a NF-κB-Mediated Mechanism

**DOI:** 10.1371/journal.pone.0032905

**Published:** 2012-03-05

**Authors:** Yong Xu, Fang Fang, Daret K. St. Clair, William H. St. Clair

**Affiliations:** 1 Graduate Center for Toxicology, University of Kentucky, Lexington, Kentucky, United States of America; 2 Department of Radiation Medicine, University of Kentucky, Lexington, Kentucky, United States of America; University of Kentucky College of Medicine, United States of America

## Abstract

**Background:**

Prostate specific antigen (PSA) is traditionally used as an indicator for the presence of prostate cancer (PCa) and radiotherapy is generally used to treat inoperable and locally advanced PCa. However, how cellular PSA level is associated with sensitivity of PCa to radiotherapy is unknown. The previous finding that the RelB-based NF-κB alternative pathway differentially regulates PSA and interleukin-8 (IL-8) in aggressive PCa has directed our attention to the role of RelB in the response of PCa to radiotherapy.

**Methodology/Principal Findings:**

RelB and its targets PSA and IL-8 in PCa cells were manipulated by ectopic expression in PCa cells with a low endogenous level of RelB (LNCaP) and by RNAi-based knock-down in PCa cells with a high constitutive level of RelB (PC3). The effects of RelB, PSA and IL-8 on the response of PCa to radiation treatment were examined *in vitro* and in xenograft tumors. RelB regulates PSA and IL-8 in an inverse manner. When the cellular levels of PSA and IL-8 were directly modulated by genetic manipulations or by the addition of recombinant proteins, the results demonstrate that up-regulation of IL-8 enhanced radioresistance of PCa cells and concurrently down-regulated PSA. In contrast, up-regulation of PSA resulted in reduced radioresistance with concurrent down-regulation of IL-8.

**Conclusion/Significance:**

RelB plays a critical role in the response of PCa to radiotherapy and the inverse expression of IL-8 and PSA. The results identify a previously unrecognized relationship between IL-8 and PSA in the response of PCa cells to radiotherapy.

## Introduction

Prostate cancer (PCa) is the second most common malignancy in North America and the second leading cause of cancer deaths in U.S. men [1]. The serum concentration of PSA in men is used as an indicator of disease of the prostate and increased PSA level is used extensively as a biomarker of PCa. Radiotherapy is generally used to treat early-stage, inoperable and locally advanced PCa. However, whether cellular PSA could be used as indicators of tumor responsiveness to radiotherapy is unknown.

NF-κB, an inflammatory responsive transcription factor, plays an important role in tumorigenesis and resistance to therapy-induced cytotoxicity [2]. The high constitutive level of NF-κB in cancers has been implicated as a major protective mechanism against cancer treatment [3]. Thus, inhibition of NF-κB is being considered as a target for enhancing the efficacy of conventional chemotherapy and radiotherapy [4]. We have previously demonstrated that overexpression of RelB, a NF-κB member, suppressed PSA expression in androgen-responsive LNCaP PCa cells, which suggests a negative effect of NF-κB on PSA expression [5]. Serendipitously, we also have found that in animals bearing RelB-overexpressed LNCaP tumors, the circulating level of IL-8 was increased.

IL-8, a NF-κB-activated cytokine, is thought to be an important factor in tumor metastasis and resistance to treatment [6]–[9]. The circulating level of IL-8 is increased in advanced PCa at the stage when the tumor no longer responds to anti-androgen therapies [10]. Furthermore, expression of IL-8 enhances PCa androgen-independent metastasis [11], [12]. Thus, the expression of IL-8 may have significant prognostic value for PCa growth and response to therapy. The present study investigated the roles of PSA and IL-8 in the radioresistance of PCa cells using *in vitro* and *in vivo* approaches. The results reveal the previously unrecognized role of IL-8 and PSA as determinants of the sensitivity of PCa to radiotherapy.

## Results

### NF-κB up-regulates IL-8 but down-regulates PSA in PCa cells

**Figure 1:**
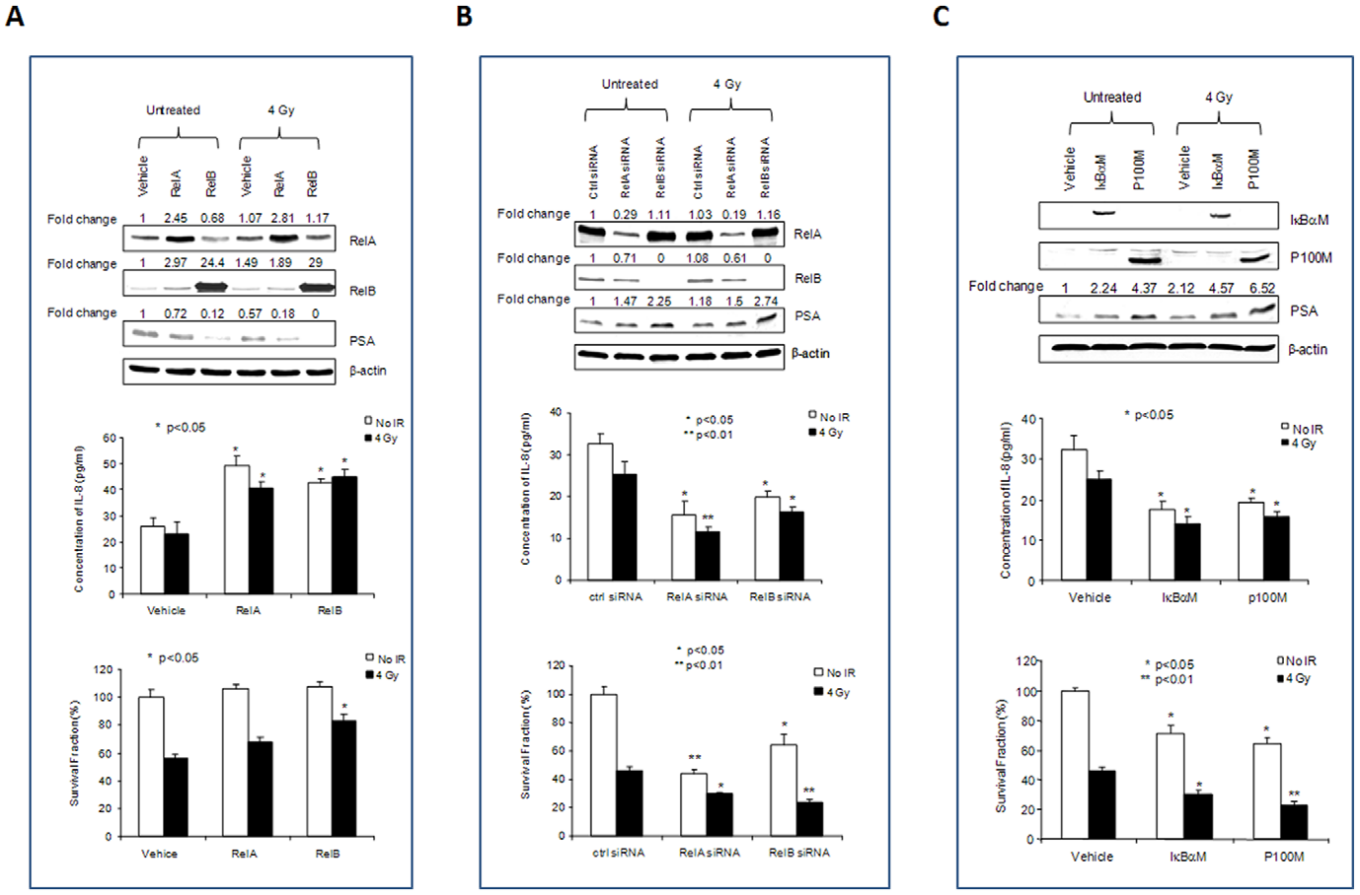
Differential regulation of PSA and IL-8 by NF-κB in LNCaP cells. To manipulate NF-κB function, RelA and RelB cDNA constructs (A), RelA and RelB siRNAs (B), and RelA and RelB nuclear translocation inhibitors (IκBαM and p100M) (C) were transfected into LNCaP cells prior to IR treatment. The levels of transfected proteins and the related target PSA were quantified by Western blots (top panel). The Western images were normalized by β-actin and then normalized by a vehicle control. Fold changes are indicated. IL-8 concentrations in media were quantified by an Elisa kit (middle panel). Cell survivals were quantified using Trypan blue exclusion assay (bottom panel). * (p<0.05) and ** (p<0.01) indicate statistical significances as compared to the vehicle control.

Activation of the NF-κB pathway is thought to be a major factor contributing to the development of the radioresistance of cancer cells. Because RelA and RelB are the two major members of the NF-κB family found in PCa cells [13], we determined the effects of RelA and RelB on IL-8 and PSA expression and radiosensitivity using multiple approaches for activation and inactivation of the NF-κB pathway. First, RelA or RelB was overexpressed in LNCaP cells by transfection with its respective cDNA constructs. As shown in Fig. 1A, the levels of PSA were decreased by both RelA and RelB when they were overexpressed in LNCaP cells. In contrast, the levels of IL-8 were increased by both RelA and RelB. The corresponding effect of RelA and RelB on radioresistance of LNCaP cells was greater with overexpression of RelB.

Second, the endogenous RelA and RelB in LNCaP cells were knocked-down using the RNA interference (RNAi) approach. RelA siRNA or RelB siRNA was transfected into LNCaP cells to silence their expression. As shown in Fig. 1B, the levels of PSA inversely correlate to the reduced levels of both RelA and RelB, with a greater effect observed for RelB. The levels of IL-8 were similarly decreased in RelA and RelB knocked-down in the cells, with a greater effect for RelA siRNA. Radiosensitivity of LNCaP cells was enhanced by reducing either RelA or RelB (Fig. 1B). The cell survival in control siRNA was reduced to 46.3% by radiation treatment, whereas the cell survival was further reduced to 30.1% or 23.4% when RelA or RelB was knocked-down. Statistical analysis showed the significant differences of RelA siRNA and RelB siRNA as compared to control siRNA. Thus, knock-down of RelB nearly doubled the cell killing effect of radiation. However, the killing of RelB siRNA plus radiation was slightly less than the combined effects of siRNA alone and radiation alone. Thus, it is possible the second hit to cells that were already dead may account for this observation. Third, RelA and RelB were inhibited by blocking their nuclear translocation to further confirm their roles in the regulation of PSA and IL-8 expressions. Mutant construct for IκBα (IκBαM) [14] or p100 (p100M) [15] was transfected into LNCaP cells to block RelA:p50 dimer or RelB:p52 dimer nuclear translocation, respectively. As shown in Fig. 1C, the responses of PSA and IL-8 expressions were similar to the results obtained from the RNAi method (Fig. 1B). Thus, blockage of either RelA or RelB nuclear translocation efficiently enhanced the radiosensitivity of LNCaP cells.

### PSA inversely correlates to IL-8 in PCa cells

**Figure 2:**
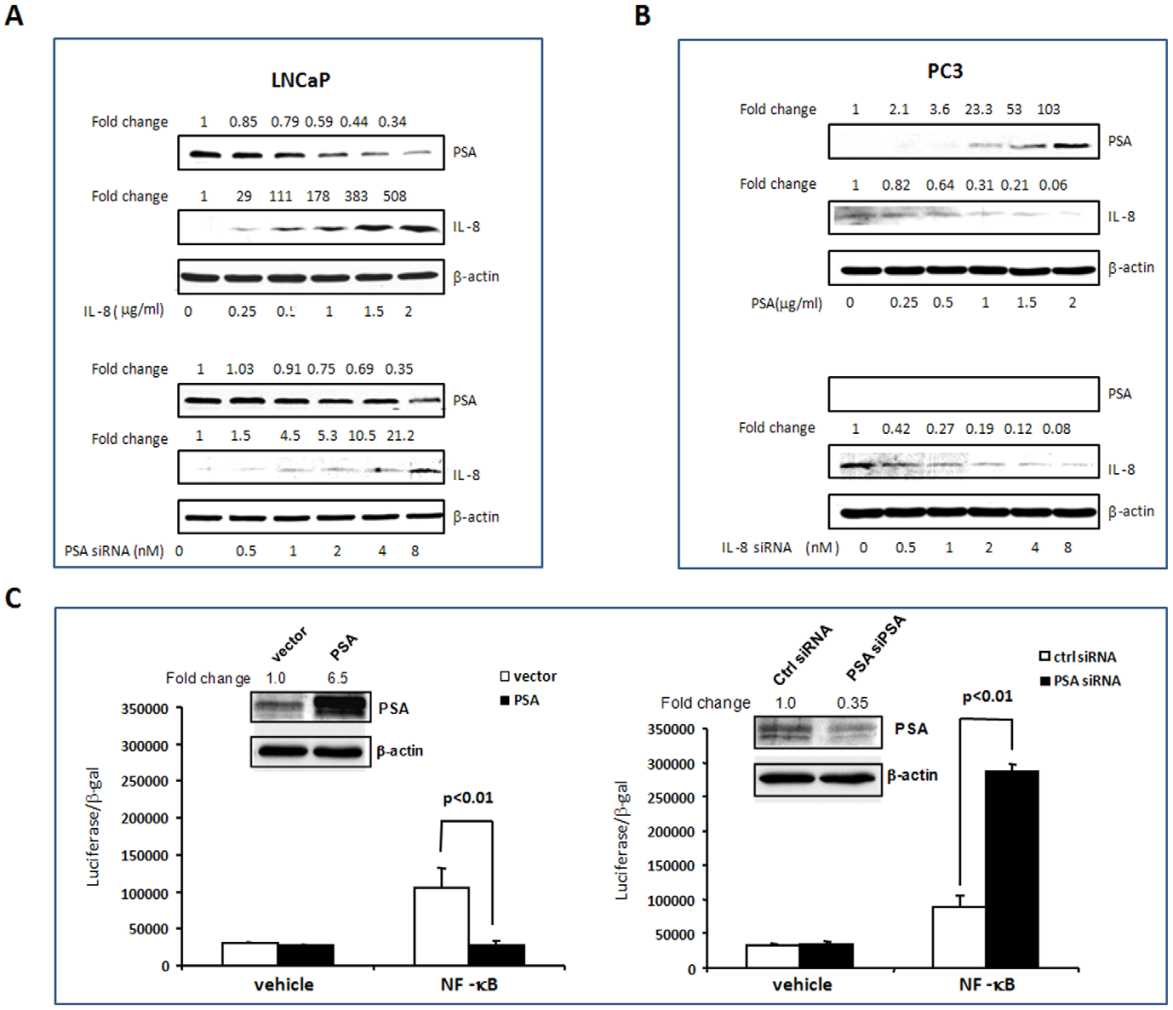
Reverse correlation of PSA and IL-8 in PCa cells. To manipulate PSA and IL-8 in PCa cells, LNCaP cells were transfected with an IL-8 cDNA construct and a PSA siRNA (A); PC3 cells were transfected with PSA cDNA construct and IL-8 siRNA (B). To test whether PSA influences NF-κB transcriptional activation, a NF-κB-luciferase reporter construct was co-transfected with a PSA expression construct or PSA siRNA and a β-gal expression construct into LNCaP cells. The luciferase responses were normalized with β-gal activities (C). PSA and IL-8 levels were quantified by Western blots with normalization as described in Figure 1.

PSA and IL-8 have been shown to be important biomarkers in multiple stages of PCa progression. We have shown that up-regulation of IL-8 but down-regulation of PSA is associated with increasing tumorigenicity of PCa cells [5]. Two PCa cell lines were used to validate the correlation of PSA to IL-8: LNCaP, an androgen responsive PCa cell line, expresses PSA and a low level of IL-8; PC3, an androgen-independent PCa cell line, expresses a high level of IL-8, but PSA is undetectable. After transfection of an IL-8 cDNA expression construct into LNCaP cells, the relative levels of PSA were quantified. As IL-8 expression increased, cellular PSA levels dose-dependently decreased. In addition, knocking- down PSA by PSA siRNA resulted in dose-dependent increases in the cellular IL-8 levels (Fig. 2A). Furthermore, expressing PSA in PC3 cells led to reduction in IL-8 production. However, there were no expressions of PSA in PC3 cells even when IL-8 was knocked- down by siRNA (Fig. 2B). It has been shown that NF-κB activation can mediate the prosurvival pathway of IL-8:CXCR2 signaling [16]. To probe whether NF-κB may be a mediator for the observed inverse relationship between IL-8 and PSA, a NF-κB-luciferase reporter construct was co-transfected into LNCaP cells with the PSA expression construct or PSA siRNA. As shown in Fig. 2C, over- expression of PSA suppressed but PSA deficiency enhanced NF-kB-mediated transcriptional activation.

### PSA and IL-8 play opposite roles in the radiosensitivity of PCa cells

**Figure 3:**
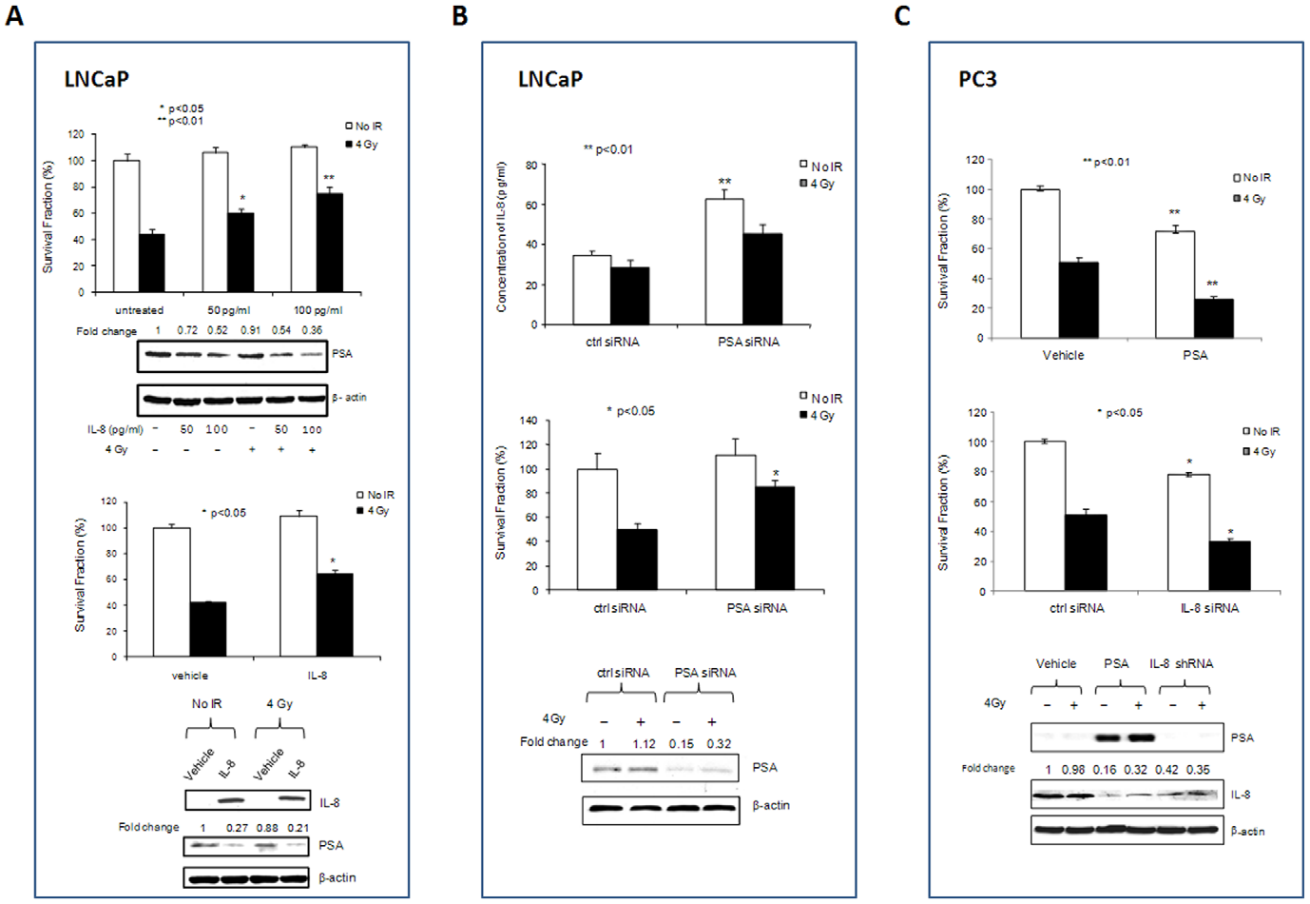
The effects of PSA and IL-8 on radioresistance of PCa cells. To determine the roles of PSA and IL-8 in radiosensitivity of PCa cells, LNCaP cells were treated with IL-8 protein or stably transfected with IL-8 cDNA (A) and stably transducted with PSA shRNA lentivirus (B); PC3 cells were stably transducted with PSA cDNA and IL-8 shRNA lentivirus (C). The levels of PSA were quantified by Western blots and the levels of IL-8 were quantified by either Western blots or an Elisa kit. Cell survival was quantified by Trypan blue exclusion assays. Western blots were normalized with controls and cell survival fraction analysis as described in Figure 1.

Being NF-κB regulated gene products, PSA and IL-8 may play an important role in the responses of PCa to radiotherapy. We therefore manipulated the levels of PSA and IL-8 to directly determine their roles in the radiosensitivity of PCa cells. Increasing IL-8 in LNCaP cells by either treating the cells with IL-8 protein (Fig. 3A, top panel) or stably transfecting IL-8 cDNA in the cells (Fig. 3A, low panel) resulted in a decrease of PSA levels and sensitivity to radiation. In addition, lentiviral-based shRNA stable knock-down of PSA in LNCaP cells resulted in increasing IL-8 levels and reducing radiosensitivity of the cells (Fig. 3B). To further verify the roles of PSA and IL-8 in radiosensitivity of PCa cells, PSA and IL-8 in PC3 cells were modulated by transfection of PSA cDNA and IL-8 siRNA, respectively. Lentivirus-based expression of PSA and knock-down of IL-8 in PC3 cells led to high levels of PSA, reduction of IL-8 levels and increased radiosensitivity of PC3 cells (Fig. 3C). Together, these results indicate the inverse relationship between IL-8 and PSA in PCa cells and suggest that PSA and IL-8 have opposite effects on the radiosensitivity of PCa cells.

### Up-regulation of RelB reduces radiosensitivity of LNCaP tumor *in vivo*

**Figure 4:**
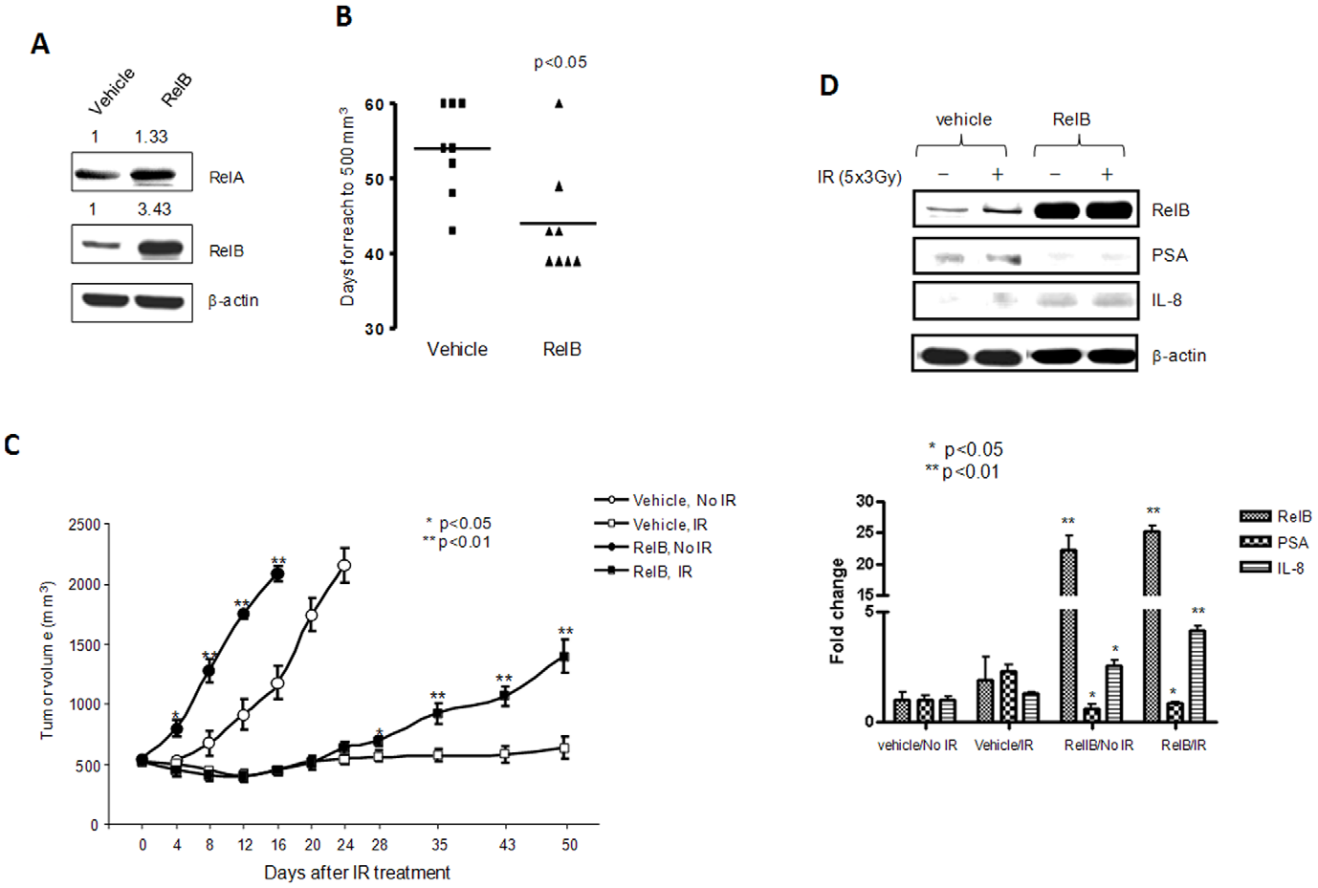
The effect of overexpression of RelB on radioresistance of LNCaP tumors. A stable RelB expressed LNCaP clone was characterized by Western blots (A). The RelB/LNCaP cells were injected into male mice for tumor formation and growth reaching to 500 mm^3^ (B). The tumors were treated IR (5×3 Gy) and tumor volumes were measured until they reached 2000 mm^3^ (C). RelB, PSA and IL-8 levels were quantified in the tumor lysates and statistically analyzed (D). Statistical significances of tumor volumes (C) and the levels of the target proteins (D) between RelB expressed and vehicle control in both no- IR and IR treated groups are indicated.

The above data obtained *in vitro* implicate RelB and it targets, IL-8 and PSA, as having an important role in the radiosensitivity of PCa. To verify the role of RelB in the radiosensitivity of PCa and the inverse relationship between IL-8 and PSA expressions *in vivo*, a stable RelB-expressing LNCaP clone was generated (Fig. 4A) and subcutaneously injected into the flanks of nude male mice; tumor growth was monitored for 50 days by measuring tumor size. Tumor growth rate change was quantified by the time (days) required for tumor size to reach a volume of 500 mm^3^. Fig. 4B shows that the average time for the control group to reach 500 mm^3^ was 54±6.7 days. Tumor growth rate increase in the RelB-expressed clone took 43±5.6 days to reach 500 mm^3^. Animals with an average tumor size of 500 mm^3^ were randomized into two groups for 1) treatment with fractionated radiation (3 Gy radiation per day for 5 days) and 2) sham controls. Mice were humanely sacrificed when tumors reached the maximum size of 2000 mm^3^. As shown in Fig. 4C, in the unradiated mice, vector control tumors grew from 500 mm^3^ to 2000 mm^3^ in 25±3.7 days, whereas the tumor expressing RelB reached the same size in 14±2.3 days. Ionizing radiation (IR) effectively prevented the growth of control tumor within the 50-day period of the experiment. However, IR only stopped the growth of RelB expressing tumors for 20 days post-radiation, after which tumor regrowth was observed. The experiment was terminated because some mice in the radiated groups developed poor health conditions implicated by clinical signs of systemic disease such as dehydration, labored or shallow breathing, and losing 20% of their maximum weight. After the tumors were removed, the expression of RelB and levels of PSA and IL-8 in the tumor tissues were quantified using an appropriate antibody for each protein. Consistent with the high levels of RelB identified in the RelB-expressed tumors, IL-8 was increased but PSA was decreased in the same tumor tissues (Fig. 4D).

### Down-regulation of RelB enhances radiosensitivity of PC3 tumors

**Figure 5:**
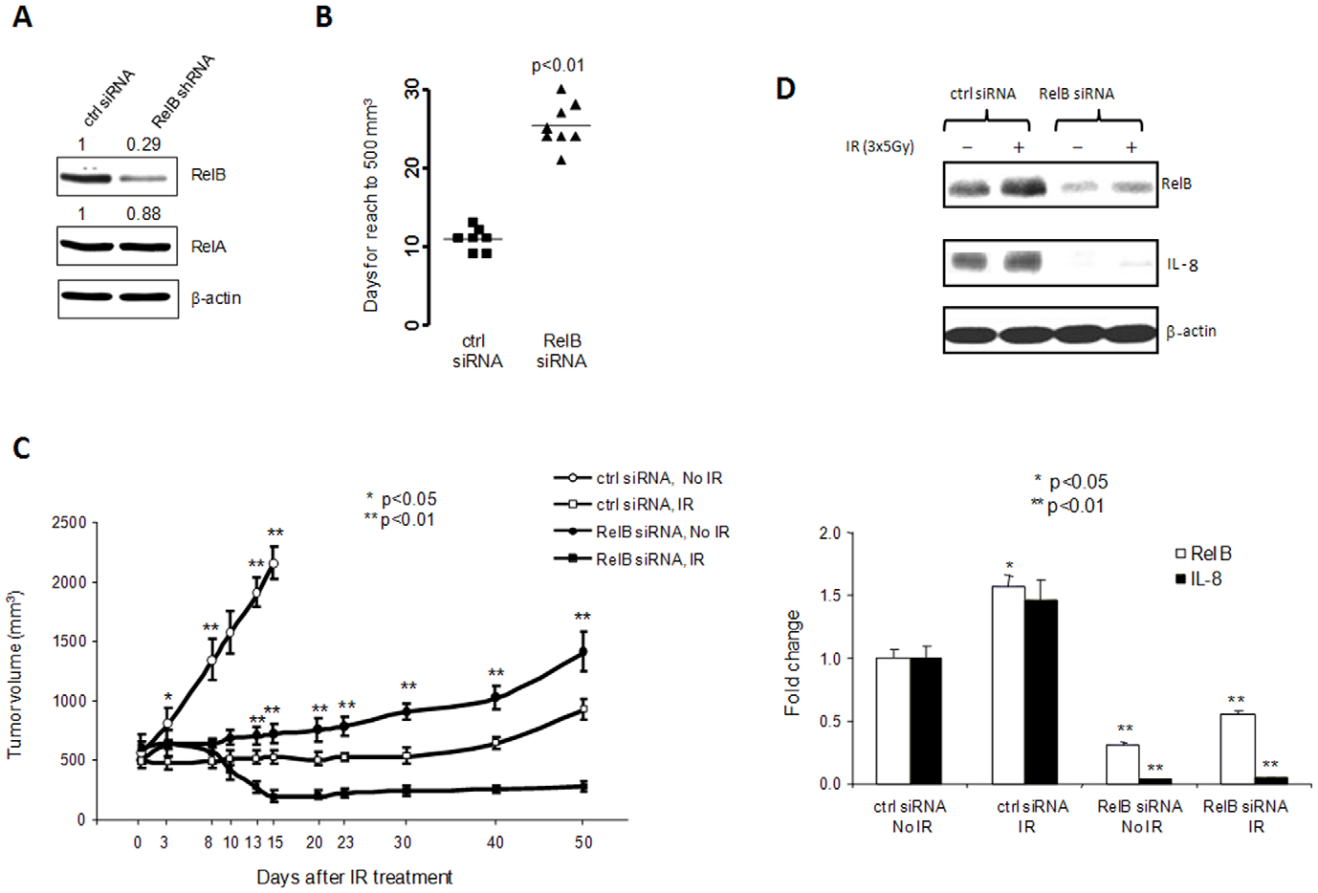
The effect of knock-down RelB on radioresistance of PC3 tumors. A lentivirus-based RelB siRNA knock-down PC3 clone was characterized by Western blots (A). The RelB shRNA/PC3 cells were injected into male mice for tumor formation (B). The tumors were treated with IR (3×5 Gy) when tumor volumes reached 500 mm^3^ and tumors were allowed to grow to 2000 mm^3^ (C). RelB and IL-8 levels were quantified in the tumor lysates and statistically analyzed (D). Statistical analysis for tumor volumes and the levels of target proteins as described in Figure 4.

To further validate the protective role of RelB against radiation, a lentivirus-based RelB siRNA PC3 clone was selected (Fig. 5A) and injected into the flanks of nude male mice. In the control siRNA group, the average time for a tumor to reach 500 mm^3^ was 11±2.1 days, whereas the average time in the RelB knock-down group extended to 26±5.1 days (Fig. 5B). Tumors were treated with IR at 3×5 Gy after tumor reached an average size of 500 mm^3^. In unradiated control mice, the tumor size in the control siRNA group quickly grew from 500 mm^3^ to 2000 mm^3^ within 15±3.1 days. Compared to the control siRNA group, tumor growth in the RelB knock-down group was significantly slower than in the control siRNA group. IR inhibited the growth of the control tumor with the trend to regrowth after 20 days post IR. Interestingly, IR not only controlled tumor regrowth, but it also reduced tumor size in the RelB siRNA group (Fig. 5C). The differences observed *in vivo* are not simple reflections of growth differences *in vitro* because there are no significant decreases in cell growth when RelB was stably knocked-down in PC-3 cells. Thus, the radiosensitization effect by knock-down of RelB is due to inhibition of radiation-inducible NF-κB activation. The effects of siRNA-mediated knock-down of RelB were confirmed by Western blot analyses of RelB and IL-8 in the tumor tissues (Fig. 5D). These results support the role of RelB in the response of PCa to radiotherapy.

## Discussion

PSA is an androgen receptor (AR)-regulated serine protease produced by prostate epithelial cells [17]. Although an elevated serum PSA level correlates with the presence of PCa, many factors, such as inflammation, infection, prostatic hyperplasia as well as tissue processes, lead to certain loss of integrity of the prostatic gland and leakage of PSA from the lumen to the interstitial liquid and into the general circulation. [18], [19]. In addition, PCa can be present in the absence of elevated serum PSA levels [20]. Indeed, there are no detectible levels of PSA in androgen-independent PCa cell lines, such as PC3 and DU-145. Although PSA is used as a biomarker in the diagnosis of PCa, its biological function has not been fully elucidated. The results from the present study demonstrate that cellular PSA level may be an important indicator of PCa cell response to IR. Down-regulation of PSA in androgen responsive LNCaP cells leads to reduced radiosensitivity and expression of PSA in androgen-independent PC3 cells results in enhanced radiosensitivity, suggesting that PSA plays an important role in the response of PCa cells to radiotherapy.

IL-8, a pro-inflammatory cytokine, uniquely expresses in androgen-independent PCa cells but not in androgen-responsive PCa cells [8], [21]. Elevated IL-8 levels in advanced PCa have been considered to be a major factor contributing to cancer cell proliferation and survival after treatments [6], [12]. The present study reveals an interesting relationship between PSA and IL-8 in the response of PCa cells to radiotherapy. The inverse relationship between PSA and IL-8 expressions confounds a conclusion that the observed changes in radiosensitivity of PCa are dependent on PSA level; alternatively, the observed effect of PSA may be mediated, in part, by a change in IL-8 level. The data presented in this report directly demonstrate the role of IL-8 in the radiosensitivity of PCa cells. Together, these results highlight the intricate relationship between IL-8 and PSA, which contribute to the response of PCa cells to radiotherapy.

NF-κB is constitutively active in many cancers, and it functions in the up-regulation of antiapoptotic and oncogenic genes [10], [22], [23]. Another important biological function of NF-κB is related to the coordination of the innate and adaptive immune responses that maintain cellular defense systems [3]. Inhibition of NF-κB is therefore considered a useful complement to traditional chemo- or radio-therapy. NF-κB consists of five Rel-related proteins, which contain the RelA/p50 heterodimer-mediated classical pathway and the RelB/p52 heterodimer-mediated alternative pathway [3], [10], [13]. RelB is uniquely expressed in advanced PCa cells and the RelB-based alternative pathway plays an important role in the tumorigenesis of PCa [24], [25]. We have previously shown that selective inhibition of the alternative pathway remarkably suppresses the tumorigenicity of PCa cells [5], [26], [27]. The results from the present study demonstrate that RelB may also play a critical role in the radioresistance of PCa cells, in part, via an inverse effect on the expressions of IL-8 and PSA.

AR activity is essential for PCa initiation and progression. Even when PCa progresses to hormone-refractory stages, AR signaling remains through a variety of androgen-independent mechanisms [28]. Thus, ablation of AR function is the goal of first-line therapy. Although these treatments are initially effective, recurrent tumors arise with restored AR activity [29] or even with other ligand binding receptors [30]. As an AR target, PSA screening monitors the management of PCa. However, the presence of AR is not adequate for predicting the efficiency of therapy because the level of PSA may be also regulated by many other factors. A large number of studies have demonstrated that both AR and NF-κB signaling are important factors in the progression of PCa. The restoration of AR activity in the absence of androgen is considered a mechanism for PCa as it progresses to the hormone-refractory stage [29]. In addition, NF-κB-mediated IL-6-stat3 activation is thought to be another important mechanism for tumor metastasis [31]. However, cross talk between the two signal pathways is elusive. The results of this study demonstrate that overexpression of RelB increased the cellular level of IL-8 but decreased the level of PSA, suggesting that NF-κB target genes may be differentially regulated by different members of the NF-κB family or that RelB may serve as co-activator for IL-8 transcription while serving as a negative co-regulator for PSA transcription. It has been shown that the combination of RelB and aryl hydrocarbon receptor (AhR) can activate transcription from a RelB/AhR element uniquely located upstream from the IL-8-κB site [32]. It has also been shown that NF-κB suppresses PSA transcription through an androgen receptor element located in the promoter region of a gene [33]. Our finding is consistent with the possibility that NF-κB not only has the capacity to activate its target gene but also to inhibit its target gene depending on the composition of factors affecting transcription.

Radiation therapy causes growth arrest and death in cancer cells either by direct ionization of DNA or by generation of reactive oxygen species (ROS) that can damage the DNA. It has been shown that genotoxic agents, including IR, induced by double-strand breaks (DSBs) stimulate the NF-κB pathway to generate positive feedback loops via cytokine production, which in turn activates DNA repair mechanisms [34]–[36]. The cytokine-activated NF-κB pathway can also lead to induction of antioxidant enzymes, which protect cancer cells against ROS generating therapeutics. Our previous studies, which demonstrate that inhibition of NF-κB and its antioxidant target, manganese superoxide dismutase (MnSOD), remarkably increase radiosensitivity of aggressive prostate cancer cells [5], [25], are consistent with the finding for this later mechanism.

The mechanism by which PSA may modulate cellular response to radiation therapy remains unknown. It is possible that the observed negative effect of PSA is due, in part, to the increase in IL-8 level when PSA is reduced. Indeed, reverse association of PSA and IL-8 was observed in both androgen-responsive and androgen-independent PCa cells, suggesting that the increased ratio of IL-8 to PSA contributes to radioresistance of PCa cells. Future study to determine how the level of PSA affects the production of IL-8 would be interesting.

In summary, the present study uses complementary experimental approaches to show that RelB contributes to radioresistance of PCa cells and inversely regulates IL-8 and PSA expressions. The inverse relationship between IL-8 and PSA levels is predictive for the response of PCa cells to radiation, suggesting the potential to enhance radiotherapy of PCa by direct modulation of IL-8 and/or PSA levels. This is the first experimental evidence to implicate the role of PSA in the radiotherapy of PCa cells. Insights obtained from the present study provide a scientific basis for the use of IL-8/PSA ratio as a cellular indicator for the management of PCa cells by radiation.

## Materials and Methods

### Cell culture and treatment

Human prostate carcinoma LNCaP and PC3 (American Type Culture Collection, ATCC) were grown and maintained in RPMI media with 10% fetal bovine serum. Cells were treated with IR using a 250 kV X-ray machine (Faxitron X-ray Corp.) with peak energy of 130 kV, 0.05 mm Al filter, at a dose of 0 to 5 Gy. To examine the effect of IL-8 on radiation responses, LNCaP cells were pretreated with IL-8 (BD Biosciences) at concentrations of 0 to 100 pg/ml for 12 h prior to radiation exposure. Radiosensitivity of PCa cells was quantified using cell survival fraction determined by Trypan blue exclusion assay, as previously described [26].

### Cell transfection

To express RelA, RelB, PSA, IL-8, p100M and IκBαM proteins in PCa cells, expression constructs coding these proteins were used. RelA (p65) expression construct is a gift from the laboratory of Nancy Rice (NIH); the p65 cDNA were cloned between H*ind* III – X*ba* I sites in pRC-CMV vector; RelB, PSA and IL-8 expression constructs were purchased from Open Biosystems. RelB catalog No.: MHS1010-7507797; PSA catalog No.: MHS1010-9205472 ; IL-8 catalog No.: MHS1010-58052. The cDNA were cloned between E*coR* V – N*ot* I sites in pCMV-Sport6 vector; P100M construct was provided by Dr. Shao-Cong Sun's lab at Pennsylvania State University [37]. P100M sequence was cloned between H*ind* III – X*ba* I sites in pCMV4 vector; IκBαM construct was provided by Dr. Veronique Imbert's lab, Adhésion cellulaire, Hôpital de Sainte Marguerite, Marseille, France. IκBα (S32-36A) mutant sequence was cloned at E*coR* V site in pcDNA 3.1 vector. All the vectors contain a CMV promoter and were used without any inducible condition. The stable transfected clones were selected by neomycin resistance. To knock-down endogenous RelA, RelB, PSA and IL-8 in PCa cells, the specific siRNA (Santa Cruz Biotech.) was transfected. Lentivirus-based shRNA constructs (Open Biosystems) were used to stably knock-down the targets according to manufacturer's instructions. The levels of proteins were confirmed by Western blots. A NF-κB-luciferase reporter plasmid containing four copies of NF-κB elements linked to a SV40 promoter in pGL3 vector (Promega) was previously constructed [25]. The reporter construct was co-transfected with the PSA expression vector or PSA siRNA (Santa Cruz Biotech.) and β-galactosidase (β-gal) construct into LNCaP cells to determine the effect of PSA on NF-κB transcriptional activation.

### Animals

Four-week old male NCRNU (nu/nu athymic nude) mice were purchased from Taconic (Hudson). 1.8×10^6^ PCa cells mixed with Matrigel (BD Biosciences) were injected into the right flanks of the mice. Tumor volumes were calculated weekly using a standard formula (A×B^2^×.52; A and B represent the diagonal tumor lengths). The tumor tissues were collected, and 100 µg tissues were lysed to quantify protein levels using Western blots. Animal experiment procedures were approved by the Institutional Animal Care and Use Committee of the University of Kentucky, Approval Protocol No. 01077M2006.

#### Western blot

To quantify protein levels, cell and tissue extracts were separated using SDS-PAGE, 8% (w/v) polyacrylamide gel, and then transferred onto a nitrocellulose membrane and blotted with primary antibodies against RelA, RelB, P100, IκBα, PSA, IL-8 and β-actin. A goat anti-mouse IgG-HRP conjugated secondary antibody was used to detect RelA, PSA, IL-8, and β-actin. Other proteins were detected using a goat anti-rabbit IgG-HRP conjugated secondary antibody. Immunoblots were visualized by an enhanced chemiluminescence detection system (ECL, Amersham Pharmacia Biotech.).

### IL-8 quantification

Cultured media were collected at the end of experimental procedures. The levels of IL-8 secreted from cultured cells were quantified using an IL-8 Elisa kit (BD Biosciences) according to the manufacturer's protocol.

### Statistical data analyses

Multiple independent experiments were performed for each set of data. Images in Western blots were quantified using Carestream Molecular Imaging software (Carestream Health Inc.). Statistical significance was analyzed using one-way ANOVA and Tukey's Multiple Comparison Test, followed by data analysis with Graphpad Prism.
